# Epigenetic modulation and prostate cancer: Paving the way for NK cell anti-tumor immunity

**DOI:** 10.3389/fimmu.2023.1152572

**Published:** 2023-04-05

**Authors:** Filipa D. dos Reis, Carmen Jerónimo, Margareta P. Correia

**Affiliations:** ^1^ Cancer Biology and Epigenetics Group, Research Center of IPO Porto (CI-IPOP)/RISE@CI-IPOP (Health Research Network), Portuguese Oncology Institute of Porto (IPO-Porto)/Porto Comprehensive Cancer Center Raquel Seruca (Porto.CCC), Porto, Portugal; ^2^ Master Program in Oncology, School of Medicine & Biomedical Sciences, University of Porto (ICBAS-UP), Porto, Portugal; ^3^ Department of Pathology and Molecular Immunology, School of Medicine & Biomedical Sciences, University of Porto (ICBAS-UP), Porto, Portugal

**Keywords:** NK cells, prostate cancer, epigenetics, epigenetic modulating drugs, tumor microenvironment (TME), immune landscape

## Abstract

Immunoepigenetics is a growing field, as there is mounting evidence on the key role played by epigenetic mechanisms in the regulation of tumor immune cell recognition and control of immune cell anti-tumor responses. Moreover, it is increasingly acknowledgeable a tie between epigenetic regulation and prostate cancer (PCa) development and progression. PCa is intrinsically a cold tumor, with scarce immune cell infiltration and low inflammatory tumor microenvironment. However, Natural Killer (NK) cells, main anti-tumor effector immune cells, have been frequently linked to improved PCa prognosis. The role that epigenetic-related mechanisms might have in regulating both NK cell recognition of PCa tumor cells and NK cell functions in PCa is still mainly unknown. Epigenetic modulating drugs have been showing boundless therapeutic potential as anti-tumor agents, however their role in immune cell regulation and recognition is scarce. In this review, we focused on studies addressing modulation of epigenetic mechanisms involved in NK cell-mediated responses, including both the epigenetic modulation of tumor cell NK ligand expression and NK cell receptor expression and function in different tumor models, highlighting studies in PCa. The integrated knowledge from diverse epigenetic modulation mechanisms promoting NK cell-mediated immunity in various tumor models might open doors for the development of novel epigenetic-based therapeutic options for PCa management.

## 1 Prostate cancer

Prostate cancer (PCa) is the second most commonly diagnosed cancer in men and the fifth most deadly worldwide (1). There are several well-established risk factors, such as advanced age, African origin, and family history of PCa (2). When diagnosed at early stages, localized PCa is potentially curable by local therapy such as radical prostatectomy (RP) or radiotherapy (RT). However, between 27% and 53% of patients develop prostate-specific antigen (PSA) recurrence, defined by a rising PSA level (2, 3). Regarding advanced hormone-sensitive prostate cancer, the standard treatment is androgen deprivation therapy (ADT), which consists of agents targeting the androgen pathway, such as luteinizing hormone-releasing hormone (LHRH) agonists and antagonists (3). Despite an initial response by most patients, resistance to ADT can occur, leading to castration-resistant PCa (CRPC) and metastatic castration-resistant PCa (mCRPC). CRPC can sustain androgen signaling regardless of the low levels of circulating androgens, through different mechanisms such as increased intra-tumoral hormone synthesis, androgen receptor (AR) amplification, mutations, as well as dysregulated expression of AR coactivators and corepressors. In this setting, treatment options include chemotherapy and second-generation antiandrogens, which are not curative but can increase patient survival, although all patients who are treated for mCRPC will eventually progress (3, 4). AR^low^ or AR^-^ PCa with neuroendocrine differentiation constitutes a form of aggressive AR-independent CRPC that occurs either *de novo* or through transdifferentiation, for which therapeutic approaches are limited to chemotherapy (5). This advanced form of disease poses several challenges besides the emerging mechanisms of androgen independence, such as high genomic heterogeneity, epigenetic deregulation, and a pro-immunosuppressive environment. Moreover, PCa therapies targeting angiogenesis are currently being explored. The expression of vascular endothelial growth factor A (VEGF-A), a marker of angiogenic activity, is known to be increased in PCa and associated with poorer prognosis, distant metastasis and advanced tumor grading (6–8). Clinical trials have shown some beneficial effects of targeting VEGF-A in patients with hormone-sensitive recurrent PCa, however, this was not observed in patients with castration-resistant PCa, and these therapies were also associated with adverse effects and high toxicity (8). Ongoing clinical trials intend to meet the need for new treatment strategies concerning these patients, which include immunotherapy in combination with other therapies (4).

### 1.1 Tumor immune landscape of prostate cancer

The tumor microenvironment (TME) encompasses a network of stromal fibroblasts, myofibroblasts, mesenchymal stem cells (MSCs), endothelial cells, as well as immune cells, and their secreted factors such as chemokines, cytokines, and extracellular matrices (ECMs). TME has an impact on tumor cell survival, proliferation, resistance to conventional therapies, metastatic dissemination, and evasion of immune surveillance (4, 9). Several studies have been conducted to exploit the landscape of immune cell populations of the PCa TME, their possible influence on tumor progression and prognosis, and to understand the changes that occur in prostate cancer cells that could lead to evasion from immune responses (**Table 1**). PCa is considered a “cold tumor”, with low immune cell infiltration, immunosuppressive microenvironment and infiltrating effector lymphocytes with dysfunctional phenotype (73). Recently, Wu et al. compared *in silico* RNA sequencing data from normal prostate tissue and PCa tissue, observing increased infiltration of neutrophils, activated mast cells, M1 and M2 macrophages, resting natural killer cells, resting dendritic cells, and naïve B cells in PCa tissues compared to normal tissues. However, monocytes, CD8^+^ T cells and activated mast cells showed higher infiltration in normal tissues. Notably, T cells accounted for the highest infiltrating cells in both normal and PCa tissues (40).

**Table 1:** Summary of the immune cell landscape in PCa.

Cell type	Molecules/Mechanisms	Biological relevance in prostate cancer	Source
Neutrophils	NLR	Tumor evasion of neutrophil-mediated cell killing High NLR associated with shorter OS, biochemical recurrence, high PSA and Gleason score	(10–15)
Mast cells	MMP-9	Increased in well-differentiated adenocarcinoma, but not in poorly differentiated tumors Conflicting studies regarding prognosis (Gleason score, biochemical recurrence, and development of metastases) Early-stage PCa progression dependent on MMP-9 production	(16–20)
Macrophages	MCP-1 IL-6 MST1R TREM-1 IL-10 C5a, CXCL1, CCL2 CCL5	TAM M2 phenotype, correlating with poorer patient prognosis. Higher TAM density associated with higher Gleason score, higher serum PSA, biochemical recurrence and worse DMFS M2 polarization by PCa cell-secreted MCP-1, impairing T cell proliferation and activation PCAFs release of IL-6 leads to M2 skewness Macrophage MST1R expression drives prostate tumor growth Macrophage AR signaling increases IL-10 and TREM-1 signaling, supporting PCa cell migration and invasion PCa cell CM-derived IL-10 induced an M2 phenotype Macrophage-derived C5a, CXCL1, and CCL2 increased PIN cell proliferation IL-6, induced by PCa cell-secreted BMP-6, stimulated PCa neuroendocrine differentiation CCL5 promoted PCa stem cell self-renewal and metastasis through β-catenin/STAT3 signaling	(19, 21–32)
Dendritic cells	CCR7 CD73, TNF-α, IL-12	Improved DMFS PCa cell CM inhibits CCR7 on DCs, impairing DC migration PCa-derived exosomes induce CD73 on DCs, inhibiting TNF-α and IL-12, impairing CD8^+^ T cell responses	(19, 33, 34)
MDSCs	NE STAT3/ARG1 IL-23	Significantly increased in PCa patients and correlated with elevated PSA levels and shorter OS Pro-tumorigenic role due to NE production, increasing PCa cell proliferation, migration, and invasion, as well as xenograft growth Increased phosphorylation of STAT3 in MDSCs, and upregulation of ARG1, inhibiting CD8^+^ T cell proliferation, production of IFN-γ and Granzyme-B MDSCs secrete IL-23, suggested to drive CRPC, inducing PCa cell survival and proliferation	(35–39)
T cells	CD4, CD8 FGF11/miRNA-541, MMP9 *GZMB*, Perforin, IFN-γ PD-1, PD-L1, TIM-3, CD38 Ki67 TGF-β1 FoxP3	Conflicting data regarding T cell prognostic value in PCa CD4^+^ T cells promote PCa cell invasion *via* FGF11/miRNA-541 and MMP9 signaling, possibly leading to metastases Downregulation of *GZMB*, Perforin and IFN-γ, suggesting disturbed effector function PD-1, PD-L1, TIM-3 and CD38 expression in PSA-specific CD8+ T cells, associated with cell exhaustion Low Ki67 expression in CD8^+^ T cells T cell production of TGF-β1 increases tumor growth and metastases TILs are skewed towards an immunosuppressive Treg phenotype associated with Gleason score, clinical stage, shorter OS and biochemical failure-free survival, and higher Ki67 index	(19, 21, 25, 35, 40–60)
B cells	CD20 Lymphotoxin, STAT3, IKKα	Higher intra-tumoral B cell density, which correlates with clinical progression and risk of recurrence CXCL13-recruited B cells produce lymphotoxin, activating STAT3 and IKKα, promoting CRPC development	(61, 62)
NK cells	CD56 CD57 CD107a PD-1, TIM-3, TNF-α, IFN-γ, Granzyme-B VEGF, CXCL8, Angiogenin, Angiopoietin-1 NKG2D ILT2/CD85j, NKp46, CD16 ICAM-1, NANOG, miR-296-3p	Lower NK cell frequency, especially in metastatic disease Suppressed growth and invasion of PCa cells in co-culture with NK cells Improved OS, DMFS, and longer time to castration resistance are associated with increased NK cell numbers Decreased NK activity levels with cancer stage progression Increased VEGF expression in circulating NK cells NK cell infiltrates exhibit decreased CD57 expression, compared to circulating NK cells NK cells display reduced degranulation ability, as measured by decreased CD107a expression PCa cell-derived CM increased PD-1 and TIM-3 in NK cells, impaired their degranulation capability, decreasing TNF-α, IFN-γ and Granzyme-B, and increasing production of chemokines involved in M2-like macrophage polarization and angiogenesis Decreased NKG2D expression on circulating NK cells in CRPC patients PCa cell-induced expression of ILT2/CD85j and downregulation of NKp46, NKG2D and CD16 in NK cells, leading to decreased NK cell cytotoxicity Downregulation of NKG2D following incubation with exosomes isolated from CRPC patients Downregulation of ICAM-1, by high expression of NANOG and miR-296-3p	(19, 63–72)

Neutrophils are key mediators of innate immunity and have been shown to play a role in inhibiting PCa growth *in vitro* and *in vivo*. However, as bone metastatic PCa progressed, tumors were able to evade neutrophil-mediated cell killing (10). In PCa patients, a high neutrophil-to-lymphocyte ratio (NLR) is associated with shorter OS (11), biochemical recurrence after RP, high PSA and Gleason score (12–14), compared to patients with low NLR, being considered as a valid prognostic biomarker for CRPC patients treated with second-line chemotherapy (15).

Mast cells are reportedly increased in well-differentiated (WD) PCa adenocarcinoma, but not in poorly differentiated (PD) tumors (16). The role of mast cells in PCa is still controversial, with some studies indicating that high numbers of mast cells are associated with higher Gleason score, biochemical recurrence and development of metastases after RP (17, 18), while others show improved distant metastasis-free survival (DMFS) (19). These observations might be linked to different tumor subtypes and stages, since in an *in vivo* study, initial progression of WD PCa tumors was dependent on matrix metalloprotease 9 (MMP-9) production by mast cells, which was no longer essential for post epithelial-to-mesenchymal transition (EMT) stages, where PD tumors were MMP-9 self-competent (16). Besides, intratumoral mast cells appear to negatively regulate both PCa tumor growth and angiogenesis, while peritumoral mast cells seem to contribute to PCa expansion due to production of the angiogenic factor FGF-2 (20). Additional research is still required to further clarify the role of mast cells in PCa.

Macrophages have been identified as one of the most common immune cell populations in PCa (74, 75). These cells can be skewed towards a tumor-suppressive M1 phenotype or to a tumor-promoting M2 phenotype. In PCa, tumor associated macrophages (TAMs) mainly display an M2 phenotype, which correlates with more aggressive disease and poorer patient prognosis (21–23). Moreover, higher TAM density has been associated with higher Gleason score, higher serum PSA, biochemical recurrence, and worse DMFS (19, 24, 25). M2-induced polarization in PCa has been associated with production of monocyte chemotactic protein-1 (MCP-1), leading to a consequent impairment of T lymphocyte proliferation and activation (21), and also with the production of IL-6 by prostate carcinoma-associated fibroblasts (PCAFs) and PCa cells (26). In a recent study, loss of prostate epithelial macrophage stimulating 1 receptor (MST1R) was shown to suppress M2 marker expression, while epithelial MST1R activation upregulated its own expression in macrophages, in a positive feedback mechanism, driving prostate tumor growth (27). Also, AR signaling was demonstrated to upregulate Interleukin-10 (IL-10) and Triggering Receptor Expressed on Myeloid cells-1 (TREM-1) signaling on macrophages, supporting PCa cell migration and invasion (28). In addition, IL-10 induced macrophages toward an M2 phenotype, after incubation with PCa cell conditioned media (CM) (29). Macrophage-secreted molecules have also been associated with PCa development, such as C5a, CXCL1, and CCL2, by increasing prostatic intraepithelial neoplasia (PIN) cell proliferation (30). IL-6, induced by PCa cell-secreted bone morphogenetic protein-6 (BMP-6), stimulated PCa neuroendocrine differentiation (31), and CCL5 promoted PCa stem cell self-renewal and PCa metastasis through activation of β-catenin/STAT3 signaling (32). Thus, further characterization of the TAM population, and development of new treatment strategies targeting its phenotype and function, are key for PCa patient treatment.

Dendritic cells (DCs), as professional antigen presenting cells (APCs), are crucial to activate T cell responses through antigen presentation *via* major histocompatibility complex (MHC) (33). DCs have an important role in PCa immunity, conferring improved DMFS (19). However, PCa cell-derived CM has been shown to inhibit CC chemokine receptor-7 (CCR7) expression on maturing DCs, impairing chemotactic movement of DCs (33). PCa-derived exosomes are also capable of inducing CD73 expression on DCs, inhibiting Tumor Necrosis Factor (TNF)-α and IL-12 production, ultimately impairing CD8^+^ T cell responses (34).

Circulating and intra-tumoral myeloid-derived suppressor cells (MDSCs) are significantly increased in PCa patients compared to healthy controls (35, 36). In PCa, MDSCs have been suggested to have a role in establishing an immunosuppressive microenvironment and promoting cancer progression, as increased frequencies of MDSCs correlated with elevated PSA levels and shorter median OS (35), and MDSC depletion significantly decreased tumor xenograft growth/progression (37, 38). Neutrophil elastase (NE), a serine protease, might be involved in the pro-tumorigenic role of MDSCs in PCa since a small-molecule inhibitor of NE was able to significantly decrease PCa cell proliferation, migration, and invasion, as well as tumor xenograft growth (37). Circulating MDSCs from PCa patients showed increasingly activated signal transducer and activator of transcription 3 (STAT3), an immune checkpoint regulator, contributing to arginase-1 (ARG1) upregulation and consequent decrease of CD8^+^ T cell proliferation, production of Interferon-γ (IFN-γ) and Granzyme-B. Targeting STAT3/ARG1 signaling of PCa patient-derived MDSCs abrogated their immunosuppressive effects (39). Moreover, MDSCs secrete IL-23, shown to be increased in blood and tumor samples of CRPC patients, and suggested to contribute to CRPC development due to AR pathway activation, leading to PCa cell survival and proliferation in androgen deprived conditions. Inhibition of IL-23 was able to restore ADT sensitivity in mice (36).

The role played by tumor infiltrating lymphocytes (TILs) in PCa is still ambiguous and further research is necessary to clarify their prognostic value in PCa patients. While a high CD8^+^ TIL density in PCa has been independently associated with improved OS, PCa‐specific survival (41, 42) and longer time to biochemical recurrence in patients with seminal vesicle invasion (43), other studies have found an association between high CD8^+^ T cell density, worse clinical progression and PCa malignant degree (40, 44, 45). A recent study of 1567 retrospective radical prostatectomy samples with long-term metastasis and survival outcomes showed that both CD8^+^ and CD4^+^ T cells appear to be associated with worse DMFS (19). In addition, infiltrating CD4^+^ T cells were suggested to promote PCa metastasis *via* modulation of fibroblast growth factor 11 (FGF11)/miRNA-541 signaling, suppressing androgen receptor signals, which could then increase matrix metallopeptidase 9 (MMP9) expression and promote PCa cell invasion (46). The functional status of TILs is key for their cytotoxic and anti-tumor immune responses. PCa tissues obtained from RP displayed almost no expression of Granzyme-B (*GZMB*) and Perforin 1 (*PRF1*), markers of cytolytic activity, indicating that T cells infiltrating prostate tumors are functionally impaired (21). This is in agreement with another study reporting downregulation of Perforin and IFN-γ on tumor infiltrating lymphocytes, showing signs of disturbed effector function, and expression of molecules associated with lymphocyte exhaustion, such as the programmed cell-death-1 (PD-1) receptor and its ligand, PD-L1 (47–49). Moreover, PSA-specific CD8^+^ T cells of PCa patients showed higher expression of exhaustion and activation markers, T cell immunoglobulin and mucin domain 3 (TIM-3) and CD38, respectively, indicating that PSA-specific T cells are exhausted (50). Additionally, CD8^+^ T cells accumulated in PCa pelvic node metastases exhibited decreased proliferation, with low Ki67 expression (51). Cytokine production by T cells can also contribute to immunosuppression, as ablation of tumor growth factor β1 (TGF-β1) in T cells (not Treg) enhanced tumor T cell cytotoxic activity in mice, inhibiting tumor growth and metastases, and suggesting that tumor immunosurveillance escape is dependent on TGF-β1 production by conventional T cells (52). Furthermore, PCa tumors have been described to be surrounded by clusters of CD25^+^ and FoxP3^+^ regulatory T cells (Tregs) (48). These cells have a suppressive role on innate and adaptive immunity and are significantly increased in the peripheral blood and tumor tissues of PCa patients, especially in metastases, which present a higher abundance of FoxP3^+^ Tregs when compared to primary lesions (35, 53–55). In PCa, a high proportion of TILs appear to be skewed towards a Treg phenotype (FoxP3^+^) (56). Treg tumor infiltration has prognostic value in PCa patients, since higher numbers of Tregs are positively correlated with Gleason score and clinical stage (57, 58), shorter OS and biochemical failure-free survival (25, 59, 60), and higher tumor cell Ki67 index (59).

Regarding B cells, a cohort of PCa paraffin-embedded radical prostatectomy specimens displayed higher B cell density in tumor areas compared to extra-tumoral ones, which correlated with clinical progression and higher risk of recurrence (61). A study with PCa murine models reported that B cells recruited by the chemokine CXCL13 produce lymphotoxin, which then activates STAT3 and IκB kinase α (IKKα), promoting CRPC development (62).

Innate Lymphoid Cells (ILCs) are characterized by the lack of rearranged antigen receptors, recognizing tumor cells in an antigen-independent manner. They are composed by the ILC1, ILC2 and ILC3 subgroups (76, 77), and are generally known to be specialized cytokine producers, mirroring the T helper subtypes. Interestingly, ILC2 cells were found to be enriched in the peripheral blood of PCa patients, compared to healthy donors, and associated with advanced tumor stage (77).

Natural Killer (NK) cells are innate lymphocytes, belonging to the ILC1 subgroup. Contrarily to the other ILCs, they rather resemble CD8^+^ T cells, being cytotoxic and specialized in targeting infected or transformed cells, such as tumor cells. In the next chapter, we will focus on the role of NK cells in prostate cancer (78).

### 1.2 NK cells and prostate cancer

NK cell recognition of target cells relies on the expression of an array of activating and inhibitory receptors that interact with specific ligands on the surface of the target cells, resulting in killing if the stimulatory signals outweigh suppressive signals (78). Kärre and Ljunggren uncovered the missing-self mechanism, by which NK cells can distinguish healthy cells from target cells lacking MHC-I molecules, also known as Human Leukocyte Antigen Class-I (HLA-I) in humans (79). HLA-A, B and C molecules act as inhibitory ligands that bind to Killer-Cell Immunoglobulin-Like Receptors (KIRs), and HLA-E is recognized by the NKG2A transmembrane receptor expressed on the surface of NK cells. Besides the lack of MHC class I, infected or transformed cells (over)express specific ligands that are recognized by specific NK cell activating receptors. NKG2D, DNAM-1 and the natural cytotoxicity receptors (NCRs) NKp46 (*NCR1*), NKp44 (*NCR2*) and NKp30 (*NCR3*), are recognized as major activating NK cell receptors (80). UL16-binding protein (ULBP), MHC class I-related chain A (MICA) and B (MICB) are NKG2D ligands, commonly overexpressed on tumor cells. DNAM-1 recognizes the activating ligands Nectin-2 (CD112) and Polio Virus Receptor (PVR, CD155), capable of increasing NK cell triggering (80). While NCR ligands have not been completely identified in tumor cells, B7-H6 has been described as an activating tumor cell ligand recognized by NKp30 (81). Another key activating receptor is the Fcγ receptor CD16, able to bind to the Fc portion of Immunoglobulin G (IgG) antibodies in target cells, leading to antibody-dependent cell-mediated cytotoxicity (ADCC). When activated, NK cells induce target cell apoptosis *via* exocytosis of cytolytic granules containing Granzyme-B and Perforin, also releasing effector cytokines such as IFN-γ and TNF-a, and various chemokines that allow for recruitment and communication with other immune cells (78, 82). Importantly, the frequency of circulating NK cells has been found to be significantly lower in PCa patients than in healthy controls, and further reduced in metastatic disease than in localized PCa (63). Moreover, in metastatic PCa patients, increased circulating NK cell numbers confer improved overall survival and longer time to castration resistance (64), and higher intratumoral NK cell numbers have been shown to confer longer distant metastasis-free survival (19). Evidence shows that NK cell function is inhibited at multiple levels in the PCa TME (79). Different studies reported suppressed growth and invasion of PCa cells when co-cultured with NK cells, highlighting the crucial role of NK cells in prostate cancer immunosurveillance (65, 66). Moreover, NK cell activity levels in tumors gradually decrease according to stage progression (67). NK cell infiltrates from PCa tumors display decreased CD57 expression compared to circulating NK cells, associated with an immature phenotype (68). Moreover, reduced degranulation capacity against K562 cells was observed in circulating NK cells from PCa patients, compared to healthy donors (69). A recent study reported that NK cells exposed to CM of different PCa cell lines presented increased expression of the exhaustion markers PD-1 and TIM-3, and displayed impaired degranulation capability, with reduced production of TNF-α, IFN-γ and Granzyme-B. These cells also displayed increased production of chemokines involved in monocyte recruitment and M2-like macrophage polarization (69). Moreover, circulating NK cells isolated from PCa patients have shown increased expression of VEGF, a marker of angiogenic activity, as well as upregulation of CXCL8, associated with inflammation and angiogenesis. Additionally, NK exposure to PCa cell line CM increased production of the pro-angiogenic factors Angiogenin and Angiopoietin-1 (69). Co-culture experiments led to decreased recognition of PCa cells and lower NK cell cytotoxicity due to increased expression of the NK cell inhibitory receptor ILT2/CD85j (68), which recognizes HLA-A, B, C and G molecules (80), and reduction of the activating receptors NKp46, NKG2D, and CD16, by PCa cells (68). Downregulation of NKG2D on NK cells was also found following incubation with exosomes isolated from serum or plasma of CRPC patients (70). Flow cytometry analysis revealed that surface NKG2D expression on circulating NK cells was decreased in CRPC patients, compared to healthy individuals (70). PCa cells can also evade NK cell-mediated death due to high NANOG expression (a pluripotent-related transcription factor), repressing ICAM-1, a cell adhesion molecule that is necessary for the β2 integrin lymphocyte function associated antigen 1 (LFA-1)-dependent early stimulatory signal for NK cell cytotoxicity, whose low expression is correlated with a high recurrence rate in PCa patients (71, 72). ICAM-1 was also shown to be targeted by the micro-RNA 296-3p (miR-296-3p), downregulating its expression. miR-296-3p is frequently upregulated in PCa, and its knockdown in mice decreased resistance to NK cell-mediated killing, as well as PCa-derived circulating tumor cell (CTC) extravasation into the lungs and the number of pulmonary metastases (72). Thus, given the diverse mechanisms that impair NK cell cytotoxicity in PCa and their impact on patient prognosis, there is a rationale for further research on targeting NK cell-mediated immunity to treat PCa patients.

## 2 Epigenetic mechanisms involved in immune responses

Epigenetics comprises modifications, such as DNA methylation and chromatin remodeling, that do not alter the DNA sequence itself. There is growing evidence implicating epigenetic mechanisms in immune cell function and in the modulation of tumor cell recognition mechanisms (83).

DNA methyltransferases (DNMTs) catalyze the transfer of a methyl group from *S*-Adenosyl-L-methionine (SAM) to the fifth carbon of a cytosine residue, creating 5-methylcytosine (5mC) and leading to transcriptional repression (83). In addition, histone modifications play a significant role in chromatin remodeling, both in normal and neoplastic processes. Histones may undergo posttranslational modifications at the N-terminal tail, such as acetylation and methylation. Acetylation is catalyzed by histone acetyltransferases (HATs), which enables gene transcription through a decrease in DNA affinity for histones, creating an “open” chromatin conformation. Histone methylation, in which histone methyltransferases (HMTs) catalyze the transfer of the methyl group from SAM to specific lysine residues on histones, is associated with either transcriptional activation or repression, depending on the amino acid residue and the number of methyl groups added (83–85).

Epigenetic modulating agents are known to impact both cancer cells and immune cells, leading to changes in tumor cell recognition and targeting. The expression of genes part of the antigen presentation machinery, such as *HLA*, *TAP1*, *TAP2*, *B2M*, *LMP2* and *LMP7* has been upregulated upon treatment with DNMTi and HDACi (86–100). DNMT and HDAC inhibition also leads to increased expression of the co-stimulatory molecules CD40, CD80 and CD86, necessary for antigen presentation (89, 98), as well as cancer-specific antigens (101). Accordingly, dendritic cells treated with DNMTi showed increased CD40 and CD86 expression, while also secreting lower levels of IL-10 (102).

Regarding T cells, increased tumor CD8^+^ T cell infiltration has been shown in several cancer models, such as breast (96, 103), ovary (87, 104), pancreatic (105) and bladder (95) tumor-bearing mice treated with DNMTi, HDACi, EZH2i, G9a/DNMTi and LSD1i, enhancing anti-tumor immunity. Namely, epigenetic reprogramming of chemokine expression has been reported upon treatment with DNMTi, EZH2i and LSD1, increasing tumor production of CXCL9 and CXCL10 chemokines (103, 104), known to be involved in T cell recruitment to the tumor. Furthermore, increased CD8^+^ T cell Perforin expression, as well as IFNγ and TNFα production have been observed upon treatment with DNMTi (87) and HDACi (106). Moreover, PCa cell treatment with HDACi has led to increased antigen-specific CD8^+^ T cell targeting (107).

Additionally, inhibition of DNMTs and EZH2 in mice xenograft models increased CD8^+^ T to Treg ratio and decreased Treg function, leading to increased T cell activation (108, 109). Interestingly, class I/II HDAC inhibition increased Treg function and proportions in lymphoid tissues (110), while class I HDACi decreased Treg numbers and Foxp3 expression (101, 111). Additionally, EZH2 and BET inhibitors have also been described to decrease Foxp3 levels (109, 112).

Several pre-clinical studies have reported an inhibition of the immunosuppressive MDSC population upon treatment with HDACi, alone or in combination with DNMTi, indicating a reversal of the immunosuppressive TME and resulting in increased survival (100, 113–115). Another study reported that treatment with HDACi increased MDSCs within tumors, however, their immunosuppressive function was impaired, as their ability to inhibit CD8^+^ T proliferation was decreased (116). Regarding macrophages, DNMTi treatment with 5-aza in an ovarian cancer mouse model revealed an increase in the M1/M2 macrophage ratio (100).

Recently, epigenetic therapy using DNMT, HDAC and EZH2 inhibitors was shown to de-repress endogenous retroviruses (ERVs), inducing double-stranded RNA (dsRNA) that is then sensed by intracellular pattern recognition receptors (PRRs), causing a viral mimicry state that leads to increased IFN secretion and enhanced expression of IFN-stimulated genes, ultimately leading to an inflammatory environment able to recruit immune cells (117–120).

Although an improvement in treatment outcome has been observed in pre-clinical models and early clinical trial results, patients treated with different epigenetic modulating agents have shown increased expression of immune checkpoints such as PD-1/PD-L1 and CTLA-4, which could impair anti-tumor immune responses (101, 121). Thus, several clinical trials are currently focused on the potential of combining checkpoint inhibitor therapies with epigenetic drugs.

Overall, treatment with epigenetic drugs is suggested to turn “cold” tumors into inflamed “hot” tumors through several mechanisms, providing a possibility to modulate immune responses and promote anti-tumor immune targeting.

In the next chapter, we will focus on the epigenetic modulation involved on NK cell-mediated immunity, including regulation of NK cell ligands, NK cell receptors and NK cell function.

### 2.1 Epigenetic regulation of NK cell ligands

#### 2.1.1 HDAC inhibitors

Treatment of PCa cells with the class I histone deacetylase inhibitor (HDACi) valproic acid (VPA), alone or in combination with the DNMT inhibitor hydralazine, was able to upregulate MICA/B and ULBPs expression at the transcriptional level (122, 123) due to dimethylation of histone 3 at lysine 4 (H3K4me2) activating mark increase, as confirmed by chromatin immunoprecipitation (ChIP). This led to enhanced NK cell cytotoxicity against PCa cells, minimizing PCa-induced suppression of anti-tumor NK cell responses (122). Cell treatment with the HDAC inhibitor suberoylanilide hydroxamic acid (vorinostat also known as SAHA, a class I and II HDAC inhibitor), trichostatin A (TSA, a class I and class II HDAC inhibitor), or VPA led to decreased B7-H6 transcription and cell surface expression. SAHA-treated target cells in co-culture with NK cells showed impaired NKp30-dependent degranulation. The authors found that HDAC3 and the HAT cyclic adenosine monophosphate response element-binding protein (CREBBP) were required for B7-H6 expression (124).

PVR and Nectin-2 (CD112), DNAM-1 activating ligands, were shown to be upregulated upon PCa cell treatment with both HDAC inhibitors sodium butyrate (NaB) or SAHA. Besides PVR and Nectin-2, SAHA and NaB also upregulated MICA/B and ULBP1-3. This effect was more pronounced for MICA and ULBP2, whose expression increased more than 4-fold, enhancing NK cell-mediated tumor cell killing (125). Similarly, exposure of the PCa cell lines PC-3 and DU-145 to the class I HDAC inhibitor entinostat was able to upregulate MICA, MICB, ULBP1/2/5/6 and PVR expression, as assessed by flow cytometry (126). This is agreement with another study, which revealed that treatment of Ewing sarcoma and rhabdomyosarcoma cell lines with entinostat increased the expression of these ligands, however HLA expression was also increased (127). Treatment of colon carcinoma cells with entinostat led to enhancement of acetylated histone 3 (AcH3) binding to *MICA* and *MICB* promoters, increasing their expression in a dose and time-dependent manner, resulting in higher NK cell-mediated tumor cell lysis, as compared to untreated cells. Of note, entinostat treatment did not induce higher NKG2D ligand expression in normal cells (128).

Panobinostat, a pan-HDACi, was able to increase the transcription and surface expression of MICA and ULBP2 in pancreatic cancer cell lines, resulting in a more potent NK-mediated killing of panobinostat treated cells than untreated cells. Xenograft mouse models further demonstrated that enhanced NK cell-mediated tumor surveillance was NKG2D ligand-dependent (129). Panobinostat also upregulated expression of the activating ligands CD80, CD86, and Nectin-2 in tumor cells, promoting NK cell cytolytic effect (130).

Treatment of lung cancer cells with different selective HDACi revealed that only FK228, an HDAC1/2 inhibitor, induced MICA, MICB and ULBP1/3 expression, which led to increased susceptibility to NK cell-mediated lysis. However, it is important to note that FK228 also increased MHC-I inhibitory ligand expression by 1.3-fold (86). Similarly, treatment with Nexturastat A (NextA), an HDAC6i, also increased MHC-I expression, which was further increased in combination with 5-azacytidine (5-aza), a DNMT inhibitor already approved for treatment of high-risk myelodysplastic syndrome (MDS) (100).

**Figure 1:**
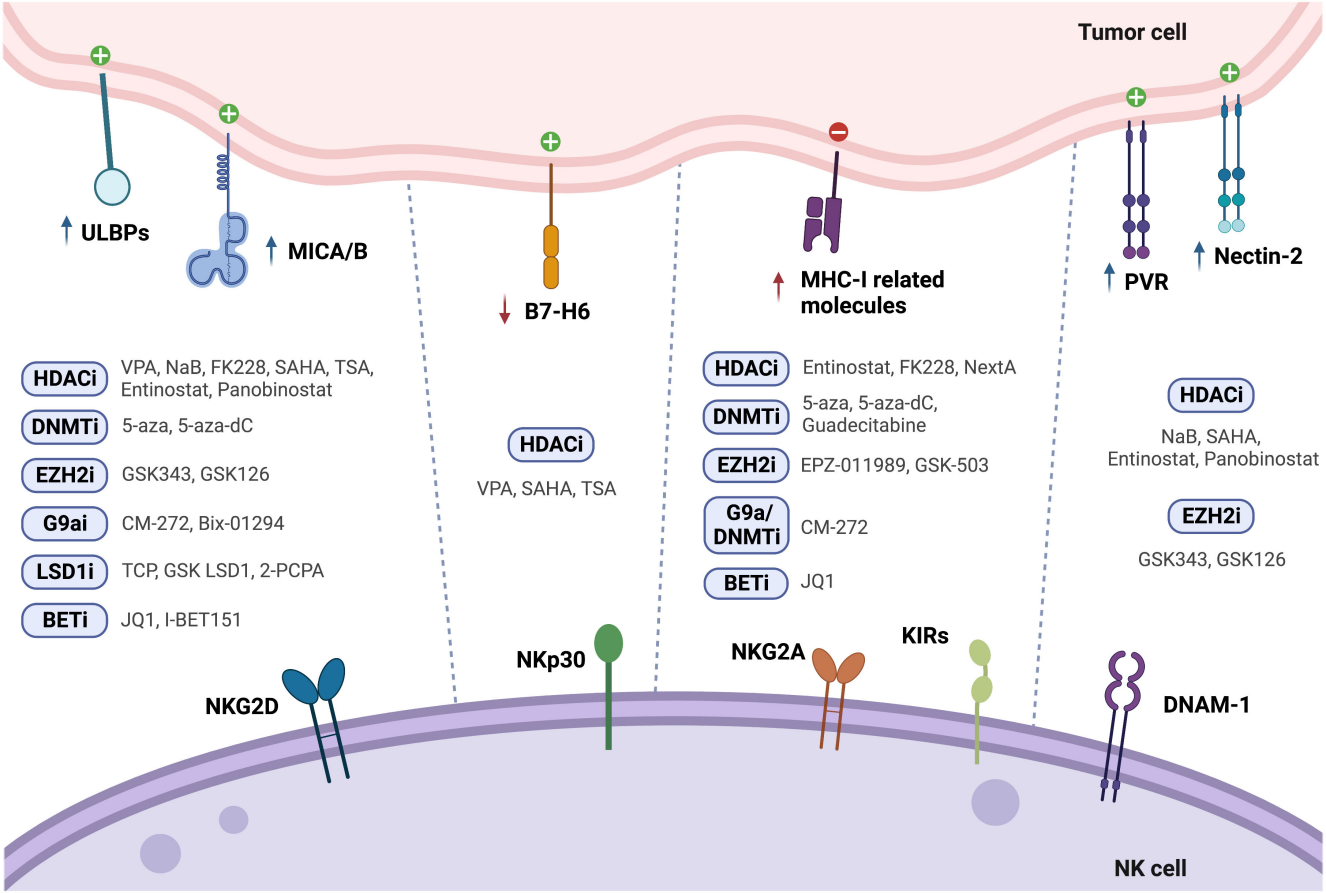
Modulation of NK cell ligands following tumor cell treatment with epigenetic drugs (↑ - upregulation; ↓ - downregulation).

HeLa cells treated with different doses of TSA showed increased ULBP1 mRNA and protein levels, as confirmed through real-time PCR and flow cytometry, when compared to untreated cells, leading to enhanced NK cell cytotoxicity. ULBP1 was shown to be repressed by transcription factor Sp3-mediated HDAC3 binding to the *ULBP1* promoter, while TSA treatment interfered with this association, decreasing HDAC3 recruitment and increasing ULBP1 expression (131). In agreement with these observations, Rossi et al. also showed TSA-induced upregulation of MICA, MICB and ULBP-1/2 expression, both at the protein and mRNA level (132) (**Figure 1** and **Table 2**).

**Table 2:** Epigenetic modulating agents and their effect on NK cell ligands.

Target	Epigenetic drug	Mechanism of action	Source
Class I HDACs Class I HDACs + DNMTs	VPA VPA + Hydralazine	↑ MICA, MICB and ULBPs, leading to increased cytotoxicity of NK cells against PCa cells ↓ B7-H6	(122–124)
Class I HDACs	Entinostat	↑ MICA, MICB, ULBP1/2/5/6 and PVR ↑ HLA	(126, 127)
HDAC1/2	FK228	↑ MICA, MICB and ULBP1/3, increasing susceptibility to NK cell-mediated lysis ↑ MHC-I	(86)
HDAC6 HDAC6 + DNMTs	NextA NextA + 5-aza	↑ MHC-I, further increased in combination treatment	(100)
Pan-HDAC	TSA	↓ B7-H6 ↑ MICA and MICB ↑ ULBP1, enhancing NK cell cytotoxicity ↑ ULBP2	(124, 131, 132)
	SAHA	↓ B7-H6, impairing NKp30-dependent degranulation ↑ PVR and Nectin-2 ↑ MICA, MICB and ULBP1-3, enhancing tumor cell killing	(124, 125)
	NaB	↑ PVR and Nectin-2 ↑ MICA, MICB and ULBP1-3, increasing tumor cell killing	(125)
	Panobinostat	↑ MICA and ULBP2 ↑ CD80, CD86, and Nectin-2, promoting NK cell cytolytic effect Enhanced NK cell-mediated tumor surveillance due to increased NKG2D ligand expression both *in vitro* and in xenograft mouse models	(129, 130)
DNMTs	5-aza 5-aza-dC	↑ *MICA*, MICB and *ULBP2*, enhancing NK cell-mediated killing ↑ MHC-I	(87–92, 133, 134)
	Guadecitabine	↑ MHC-I after stimulation with IFN-γ	(96)
EZH2	GSK126 GSK343	↑ *MICA*, *MICB*, *ULBP1-6*, *PVR* and *Nectin-2*, increasing NK cell killing of HCC cells	(135)
	GSK503 EPZ011989	↑ MHC-I	(93, 94)
G9a/DNMTs	CM-272	↑ MICA, MICB and ULBP1-6 ↑ MHC-I related molecules and B2M	(95)
G9a	Bix-01294	↑ MICA and MICB	(136)
LSD1	GSK LSD1 TCP	↑ ULBP4 and MICB, leading to enhanced killing of glioma cells	(137)
	2-PCPA	↑ ULBP2/5/6, enhancing NK-cell mediated killing of AML cells *in vitro* and *in vivo*	(138)
BET proteins	JQ1	↑ MICA, increasing tumor cell recognition by NK cells due to downregulation of MICA transcriptional repressor *IRF4* ↑ MHC-I	(139, 140)
	I-BET151	↑ MICA, increasing tumor cell recognition by NK cells due to downregulation of MICA transcriptional repressor *IRF4*	(139)

↑ - upregulation; ↓ - downregulation.

#### 2.1.2 DNMT inhibitors

Treatment of HCC cells with 5-Aza-2’-deoxycytidine (5-aza-dC, also known as decitabine), a pyrimidine analogue that incorporates into DNA and inhibits DNMTs, led to increased MICB surface expression, which enhanced susceptibility to NK cell-mediated killing (133). Raneros et al. revealed hypermethylation of the NKG2D ligands *MICA*, *MICB* and *ULBP2* in acute myeloid leukemia (AML) cell lines and patients, which correlated with absence of transcription. It was further demonstrated that treatment of AML cell lines with 5-aza or 5-aza-dC was able to restore transcription of these ligands, of which ULBP2 was the most susceptible to demethylation, resulting in increased NK cell-mediated lysis of tumor cells (134).

Treatment with 5-aza-dC has shown upregulation of MHC-I surface expression in several tumor models, such as ovarian, melanoma, lymphoma, leukemia, lung, colorectal, liver and breast cancer cells (87–91), which could possibly have a negative impact on NK cell anti-tumor immunity. Moreover, Paulson et al. showed that *ex vivo* culture of Merkel cell carcinoma (MCC) tumors treated with 5-aza resulted in upregulation of *HLA-B*, which was previously shown to be downregulated in these tumors (92). In another study, breast cancer cells treated with the next generation DNMT inhibitor guadecitabine also showed upregulation of MHC-I, but only after subsequent stimulation with IFN-γ (96) (**Figure 1** and **Table 2**).

#### 2.1.3 EZH2 inhibitors

Enhancer of zeste homolog 2 (EZH2) is one of the most studied HMTs. EZH2 is the catalytic subunit of the polycomb repressive complex 2 (PRC2), responsible for the trimethylation of histone 3 at lysine 27 (H3K27me3), leading to heterochromatin formation, epigenetic gene silencing, and transcriptional repression. In this manner, EZH2 regulates cell cycle progression, proliferation and differentiation of both cancer cells and immune cells, affecting immune surveillance and tumor growth (141). EZH2 is overexpressed in PCa, particularly in mCRPC, and has been suggested to promote PCa progression (84, 142). Treatment of hepatocellular carcinoma (HCC) cells with the small-molecule EZH2 inhibitors GSK343 or GSK126 was able to upregulate several activating NK cell ligands, such as *ULBP1-6, MICA, MICB, PVR* and *NECTIN2*, which led to a significant increase in NK cell-induced cytotoxicity dependent especially on ULBP1 and MICA. Chromatin immunoprecipitation (ChIP) assays showed EZH2 recruitment to *ULBP1* and *MICA* promoters, increasing H3K27me3 repression mark levels. Moreover, methylated-DNA immunoprecipitation (MeDIP) analysis revealed higher *ULBP1* promoter DNA methylation due to EZH2-induced DNMT3A recruitment. GSK343 treatment decreased DNMT3A recruitment to the *ULBP1* ligand promoter, reducing promoter DNA methylation and increasing ULBP1 expression, further elucidating the role of EZH2 as an inhibitor of anti-tumor immune responses (135). However, pharmacological EZH2 inhibition in tumor cell lines has also been shown to upregulate cell surface expression of the NK cell-inhibitory ligand MHC-I, following treatment with GSK503 or EPZ011989 (93, 94). This dual role highlights the need for further *in vivo* research on NK cell tumor cytotoxicity following EZH2 inhibition (**Figure 1** and **Table 2**).

#### 2.1.4 G9a inhibitors

G9a, a histone methyltransferase that catalyzes H3K9me2, can be targeted with the newly developed dual G9a/DNMT inhibitor, CM-272, described to decrease H3K9me2 and m5C levels in bladder and prostate cancer cells (95, 143). Recently, we found G9a, H3K9me2 and DNMT1 protein expression to be upregulated in a cohort of CRPC patients. Herein, CM-272 was able to significantly impair proliferation and induce apoptosis of PCa cell lines, particularly of the AR-independent DU-145 cell line (143). *In vitro* assays showed increased expression of activating NK-cell ligands such as ULBP1-6, MICA and MICB after 48h of CM-272 treatment, however, concomitantly increasing inhibitory major histocompatibility genes, such as HLA-A, -B and -C, and beta-2-microglobulin (B2M). These changes in MHC-related genes were also verified in mice treated with CM-272 in combination with cisplatin (CDDP)-based chemotherapy in a bladder cancer model (95). Furthermore, tumor cell lines treated with Bix-01294, a G9a inhibitor, also showed upregulation of MICA and MICB activating ligands (136) (**Figure 1** and **Table 2**).

#### 2.1.5 LSD1 inhibitors

Histone demethylase (KDM) LSD1 activity can be targeted with catalytic inhibitors which form covalent adducts in the LSD1 demethylation site. Treatment of glioma cells with the catalytic LSD1 inhibitors tranylcypromine (TCP) or GSK LSD1 was able to increase MICB and ULBP4 expression, enhancing human NK-cell mediated lysis of the target cells (137). Furthermore, treatment of AML cells with the LSD1 inhibitor tranylcypromine (2-PCPA) hydrochloride was able to upregulate expression of ULBP2/5/6, leading to increased susceptibility to NK cell-mediated killing, both *in vitro* and *in vivo* (138) (**Figure 1** and **Table 2**).

#### 2.1.6 BET inhibitors

Bromodomain and extra-terminal (BET) proteins recognize acetylated lysine residues in N-terminal tails of histones, modulating gene expression through transcription factor and chromatin-regulating enzymes recruitment. BET small molecule inhibitors, such as the triazolo-benzodiazepine-based JQ1 and the quinolone-based I-BET151, were able to upregulate MICA expression on multiple myeloma cells, due to downregulation of the target gene *IRF4*, a transcriptional repressor of MICA, increasing their recognition by NK cells (139). However, JQ1 has also been shown to upregulate MHC-I expression in colorectal cancer (CRC) cells (140) (**Figure 1** and **Table 2**).

### 2.2 Epigenetic regulation of NK cell receptors

#### 2.2.1 HADC inhibitors

**Figure 2:**
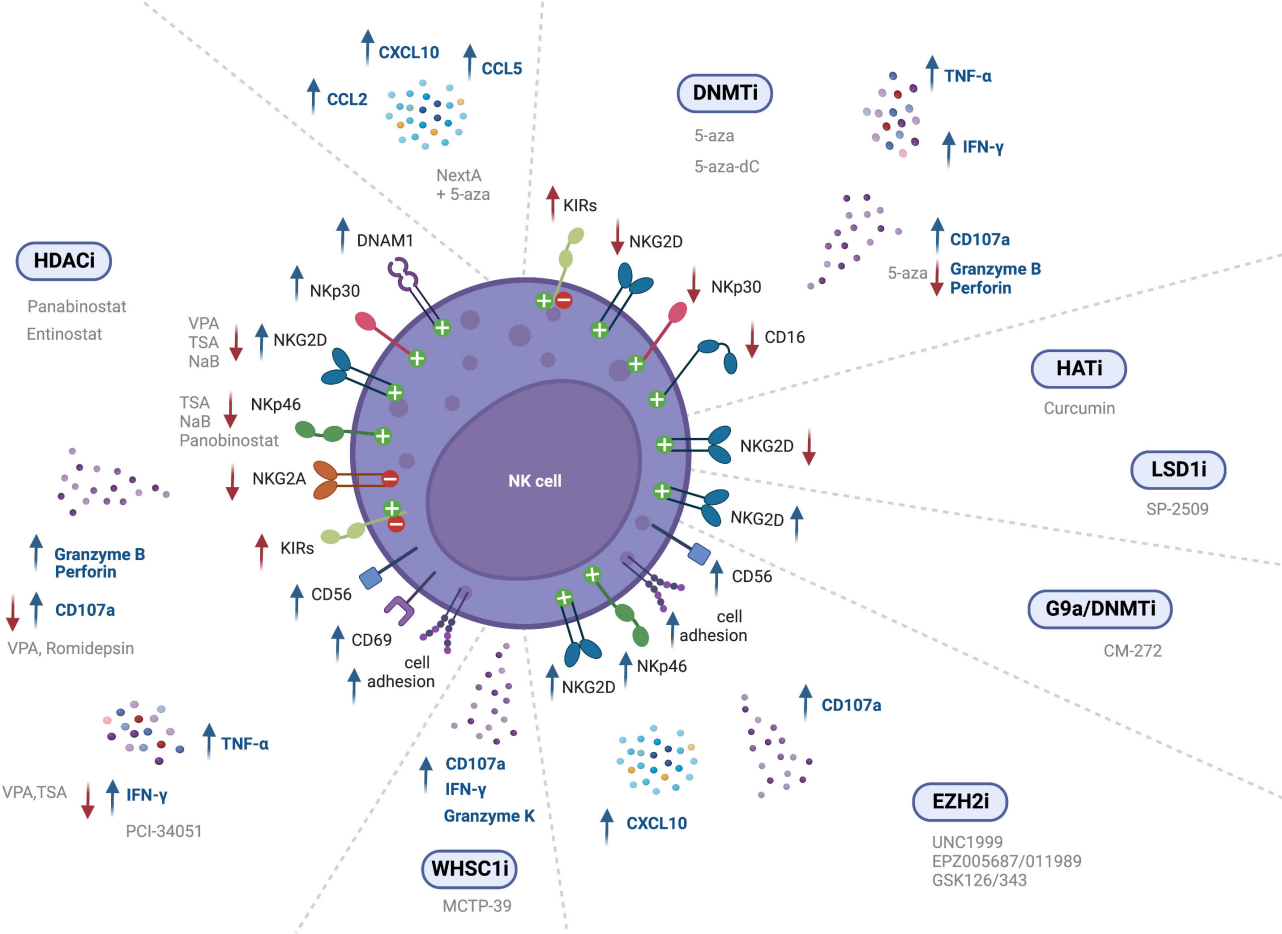
Epigenetic modulating agents and their effect on NK cell receptor expression and cell function (↑ - upregulation; ↓ - downregulation).

NK cell mediated immunity can also be modulated through epigenetic regulation of NK receptor expression. NK cell treatment with the HDAC inhibitor entinostat was shown to lead to a significant increase in activating receptors such as NKG2D (126, 128), NKp30 and DNAM-1, although resulting in a decreased expression of NKp46 (126). Furthermore, dual-treatment of NK cells and colon carcinoma cells resulted in higher NK cell mediated cytotoxicity, as compared to either entinostat-treated NK cells or entinostat-treated tumor cells alone (128). In another study, NK cells incubated with entinostat showed increased surface expression of NKG2D, although they also displayed an increase in inhibitory isoforms of KIR2DL1, KIR2DL2 and KIR2DS4, which indicates that entinostat increases not only the expression of activating receptors, but can also enhance surface expression of inhibitory receptors (127). Moreover, treatment with VPA inhibited NK cell cytotoxicity against leukemic cells due to downregulation of NKG2D, since it induced H3K9me2 and DNA methylation in the gene promoter (132, 144). NK cells stimulated with IL-12, IL-15 and IL-18, and treated with TSA or NaB also exhibited decreased NKG2D and NKp46 surface expression (132). In another study, primary NK cells showed decreased CD16, NKp46 and NKG2D expression following panobinostat treatment (145). However, a recent assessment of splenic NK cell populations from panobinostat-treated tumor-bearing mice showed increased surface expression of the activating receptors NKG2D and DNAM-1, while the expression of the inhibitory receptors NKG2A and Ly49I/C was decreased (130), albeit further mechanistic insights are still to be exploited. Panobinostat-treated NK cells showed enhanced expression of CD56, which is a marker of NK cell activation and has an important role in cell-cell contact, inducing activation signals *via* adhesion molecules (130). Also, NKG2D expression was found to be decreased upon VPA treatment through repression of STAT3 phosphorylation, following HDAC3 inhibition (146) (**Figure 2** and **Table 3**).

**Table 3:** Modulation of NK cell receptors through treatment with epigenetic modulating agents.

Target	Epigenetic drug	Mechanism of action	Source
Class I HDACs	Entinostat	↑ NKG2D, NKp30 and DNAM-1, resulting in higher NK cell cytotoxicity against tumor cells ↓ NKp46 ↑ KIR2DL1, KIR2DL2 and KIR2DS4	(126–128)
	VPA	↓ NKG2D, inhibiting cytotoxicity against leukemic cells	(132, 144, 146)
Pan-HDAC	TSA NaB	↓ NKG2D and NKp46	(132)
	Panobinostat	↓ CD16, NKp46 ↑/↓ NKG2D ↑ DNAM-1 ↑ CD56 ↓ NKG2A and LY49I/C in splenic NK cells from treated tumor bearing mice	(130, 145)
G9a/DNMT	CM-272 (+CDDP)	↑ NKG2D	(95)
EZH2	UNC1999 EPZ005687	↑ NKG2D, leading to enhanced NK cell-mediated tumor cell lysis	(147)
	EPZ011989	↑ *CCL3*, *ICAM1*, *ICAM2*, and *CD86* ↑ CD56 and NKp46	(148)
HATs	Curcumin	↓ NKG2D, impairing NK cell lytic ability	(149)
LSD1	SP-2509	↓ CD16 and NKG2D, decreasing NK cell lysis ability	(150)
JMJD3/UTX	GSK-J4	↓ NKp46 No impact in NK cell-mediated lysis of K562 cells	(151)
DNMTs	5-aza	↑ KIRs ↓ NKG2D, NKp30 and CD16	(152–154)
	5-aza-dC	↑ KIRs ↑ NKp44 ↓ NKG2D	(155, 156)

↑ - upregulation; ↓ - downregulation.

#### 2.2.2 HMT inhibitors

*In vivo* treatment with the G9a/DNMT inhibitor CM-272, in combination with cisplatin-based chemotherapy (CDDP), revealed induced expression of the activating cell receptor NKG2D (95). Treatment of NK cells with both EZH2i UNC1999 or EPZ005687 was also able to augment NKG2D expression, leading to enhanced cytolytic activity against a lymphoma cell line. ChIP analysis revealed decreased H3K27me3 at the *KLRK1* promoter, that encodes for *NKG2D* (147). Bladder cancer xenografts treated with EPZ011989, alone and in combination with cisplatin, showed greater expression of NK cell-associated genes *CCL3*, *ICAM1*, *ICAM2*, and *CD86*, indicating activated NK signaling. Moreover, immunohistochemistry (IHC) analysis revealed greater CD56 and NKp46 expression (148) (**Figure 2** and **Table 3**).

#### 2.2.3 HAT inhibitors

However, treatment with the HAT inhibitor curcumin led to hypoacetylation at the *NKG2D* gene, as shown by decreased H3K9Ac levels, downregulating *NKG2D* transcription and impairing the cytolytic ability of NK cells (149) (**Figure 2** and **Table 3**).

#### 2.2.4 KDM inhibitors

Treatment of human expanded NK cells with the LSD1 scaffolding inhibitor SP-2509, decreased CD16 and NKG2D activating receptors expression, as compared to the control. This led to reduced NK cell lysis against K562 and MOLM13 leukemic cells (150). Treatment of NK cells with GSK-J4, an inhibitor of the H3K27 demethylase jumonji domain-containing protein D3 (JMJD3/UTX), led to NKp46 downregulation, demonstrated through flow cytometry. However, co-culture assays did not show any impact in NK cell killing of K562 leukemic cells (151) (**Figure 2** and **Table 3**).

#### 2.2.5 DNMT inhibitors

Treatment of NK cells derived from healthy donors and MDS patients with 5-aza was shown to significantly increase KIR expression (152). Likewise, Gao et al. reported increased KIR expression in NK-92MI cells following treatment with 5-aza (153). Moreover, Santourlidis et al. observed induction of KIR expression on several NK cell lines following treatment with 5-aza-dC, indicating that repression of KIRs is dependent on DNA methylation (155). These results are consistent with another study showing increased surface expression of KIRs in NK cells treated with 5-aza-dC, while also observing increased NKp44 expression, and decreased NKG2D surface expression, as confirmed through flow cytometry (156). Furthermore, 5-aza treatment of NK cells resulted in a slight reduction in NKG2D, NKp30 and CD16 expression (154) (**Figure 2** and **Table 3**).

### 2.3 Epigenetic modulation of NK cell function, infiltration, and development

#### 2.3.1 HDAC inhibitors

Besides NK cell ligands and receptors, epigenetic agents may also modulate NK cell function through several mechanisms. CD107a is localized within the NK cell vesicles which contain cytotoxic granules and is brought to the cell surface upon granzyme exocytosis, thus representing a mark of degranulation. Entinostat-treated NK cells subsequently co-cultured with untreated tumor cells showed a significant increase in NK cell CD107a-positivity (127, 128). Importantly, RNA sequencing data of entinostat-treated human NK cells before co-culture showed transcriptional changes, revealing upregulation of molecules related to NK cell cytotoxic functions, such as *GZMB*, *PRF1*, *IFNG*, and *TNFA*. Thus, HDAC inhibition in NK cells led to significant transcriptional profile alterations, independently of NK cell ligand modulation or co-culture with tumor cells (127). Additionally, treatment with entinostat enhanced NK cell cytotoxicity against tumor cells (127, 128). Likewise, assessment of splenic NK cells from panobinostat-treated tumor-bearing mice also showed a significant increase in the percentage of CD107a^+^ NK cells, as well as positive for CD69, a surface marker related NK cell activation, when compared to the control. Furthermore, increased expression of cell adhesion molecules and tight junction genes was shown following panobinostat treatment, resulting in increased conjugation between NK and target cells, and subsequent enhancement of NK cell mediated-tumor cell lysis (130). In an ovarian cancer mouse model, treatment with NextA, an HDAC6 inhibitor, in combination with 5-aza led to increased expression of interferon-stimulated genes (ISGs), and of *CCL2*, *CCL5* and *CXCL10*, resulting in increased NK cell activation and infiltration into the TME (100). Moreover, selective HDAC8 inhibition with PCI-34051 increased the percentage of IFN-γ producing NK cells, as assessed by intracellular IFN-γ flow cytometry prior to co-culture with tumor cells. However, no significant changes were observed regarding NK cell killing of K562 cells (157). On the other hand, NK cell treatment with VPA impaired CD107a degranulation as well as IFN-γ secretion (132, 144). NK cells isolated from healthy donors, stimulated with IL-12, IL-15 and IL-18, and treated with TSA also exhibited decreased IFN-γ secretion, degranulation and cytotoxicity against K562 leukemic cells (132). Inhibition of NK cell degranulation was also found upon treatment of primary NK cells with panobinostat or romidepsin, resulting in decreased CD107a expression, and reduced killing of K562 leukemic cells (145). Moreover, entinostat treatment was shown to increase NK cell proliferation, as measured by Ki67 (126) (**Figure 2** and **Table 4**).

**Table 4:** Effect of epigenetic modulating agents on NK cell function, infiltration, and development.

Epigenetic drug	Target	Mechanism of action	Source
Class I HDACs	Entinostat	↑ CD107a following co-culture with tumor cells ↑ *GZMB*, *PRF1*, *IFNG*, and *TNFA* ↑ NK cell killing of tumor cells ↑ Ki67	(126–128)
	VPA	↓ CD107a ↓ IFN-γ	(132, 144)
	Romidepsin	↓ CD107a and decreased NK cell killing of K562 cells	(145)
HDAC6 + DNMTs	NextA + 5-aza	↑ ISGs, *CCL2*, *CCL5* and *CXCL10*, increasing NK cell activation and infiltration	(100)
HDAC8	PIC-34051	Increase in IFN-γ producing NK cells No significant change in NK cell killing of K562 cells	(157)
Pan-HDAC	Panobinostat	↑/↓ CD107a ↑ CD69 ↑ cell adhesion and tight junction molecules, increasing NK cell cytotoxicity against tumor cells	(130, 145)
	TSA	↓ IFN-γ secretion, degranulation, and cytotoxicity	(132)
WHSC1	MCTP-39	↑ CD107a ↑ GZMK and IFN-γ	(158)
EZH2	EPZ005687	Increased NK cell killing of K562 cells Increased NK cell differentiation, leading to a greater number of mature NK cells	(147, 159)
	UNC1999	↑ CD107a Increased Ca^2+^ influx, resulting in unbalanced vesicle release and impaired NK cell cytotoxic activity Increased NK cell differentiation, leading to a greater number of mature NK cells	(147, 160)
	GSK343	↑ CD107a Increased Ca^2+^ influx, resulting in unbalanced vesicle release and impaired NK cell cytotoxic activity	(160)
	GSK126/343	↑ CXCL10, increasing NK cell migration and suppression of tumor growth in mice	(161)
	EPZ011989	Increased frequency of tumor-infiltrating NK cells in mouse xenografts	(148)
JMJD3/UTX	GSK-J4	↓ IFN-γ, TNF-α and GM–CSF due to increase of H3K27me3 at the transcription starting site No significant change in NK cell killing of K562 cells	(151)
LSD1	SP-2509	Suppression of NK cell metabolism	(150)
DNMTs	5-aza	↑ CD107a ↑ IFN-γ ↓ Granzyme-B and Perforin, inhibiting NK cell cytolytic ability	(152, 153)
	5-aza-dC	↑ IFN-γ and TNF-α ↓ NK cell activity Increased frequency of tumor-infiltrating NK cells in mouse xenografts	(87, 154, 162)

↑ - upregulation; ↓ - downregulation.

#### 2.3.2 HMT inhibitors

Mice grafted with prostate adenocarcinoma cells treated with MCTP-39, an inhibitor of the HMT WHSC1, showed increased NK cell effector function, CD107a, Granzyme-K (GZMK) and IFN-γ expression (158). Treatment with EZH2 inhibitors has also led to increased NK function in several studies. The small-molecule EZH2 inhibitor EPZ005687 resulted in enhanced NK cell cytotoxicity against K562 cells (159). A recent study reported that NK cell treatment with the EZH2 inhibitors UNC1999 or GSK343 enhanced CD107a levels, while NK cell cytotoxicity against K562 erythroleukemia cells was unexpectedly lower than in the control group. Results indicated that EZH2i treatment upregulated the expression of the calcium channel PKD2 and increased Ca^2+^ influx, resulting in an unbalanced vesicle release, where a large number of cytotoxic particles were released to fewer target cells within a short period of time, ultimately impairing NK cell cytotoxic activity (160). A recent report revealed that treatment of hepatocellular carcinoma cells with EZH2i GSK126/343 was able to increase CXCL10 expression by tumor cells, which induced NK cell migration to the tumor site and suppressed tumor growth in a mouse model. This study also disclosed that EZH2 recruitment to the *CXCL10* promoter was dependent on HDAC10 (161). Moreover, treatment with EZH2i EPZ011989 alone and in combination with cisplatin resulted in increased frequency of tumor-infiltrating NK cells (148). Moreover, NK cell development can also be targeted with epigenetic modulating agents, since treatment of Hematopoietic Stem and Progenitor Cells (HSPCs) with the small-molecule inhibitors UNC1999 and EPZ005687 decreased H3K27me3 levels and accelerated differentiation into CD122^+^NKp46^+^ phenotype in treated cells, leading to a greater number of mature NK cells as compared to control. Thus, EZH2 inhibition is suggested to promote NK cell development *in vitro* (147, 159) (**Figure 2** and **Table 4**).

#### 2.3.3 KDM inhibitors

GSK-J4 has been shown to reduce cytokine levels in IL-15-stimulated NK cells, such as IFN-γ, TNF-α and granulocyte–macrophage colony-stimulating factor (GM–CSF), since it increased the repressive H3K27me3 mark around the transcription starting site of those genes. Despite this, GSK-J4 did not impact NK cell killing of K562 leukemic cells (151). Targeting the histone demethylase LSD1 with the scaffolding inhibitor SP-2509 was able to suppress NK cell metabolism, since treatment of NK cells derived from several donors abolished oxidative phosphorylation in NK cells, through glutathione depletion (150) (**Figure 2** and **Table 4**).

#### 2.3.4 DNMT inhibitors

NK cells from 5-aza-dC-treated mice exhibited upregulation of IFN-γ and TNF-α, which inhibited tumorigenicity *in vivo*, indicating that 5-aza-dC promotes NK cell antitumor activity (87). However, Triozzi et al. showed that, in an *in vivo* melanoma model, treatment with low-dose 5-aza-dC, either alone or in combination with IL-2, decreased NK cell activity (162). Co-culture of tumor cells with NK cells pre-treated with the DNMTi 5-aza induced higher degranulation and IFN-γ production in response to target cells, indicating increased NK cell function following 5-aza treatment (152). On the contrary, 5-aza treatment of human polyclonal NK cells has been reported to decrease Granzyme-B and Perforin secretion by NK cells, inhibiting NK cell cytolytic ability against K562 leukemic cells. The authors pose the hypothesis that this loss of NK activity may be due to excessive demethylation following 5-aza treatment (153). In a different study, although 5-aza-dC was not able to directly stimulate NK cells, it increased NK cell responsiveness to IL-2 stimulation, and induced IFN-γ production upon co-culture with tumor cells (154). An increase in the frequency of tumor-infiltrating NK cells after treatment with 5-aza-dC has also been reported (87) (**Figure 2** and **Table 4**).

## 3 NK cells, epigenetic modulation and PCa: Discussion and future perspectives

**Figure 3:**
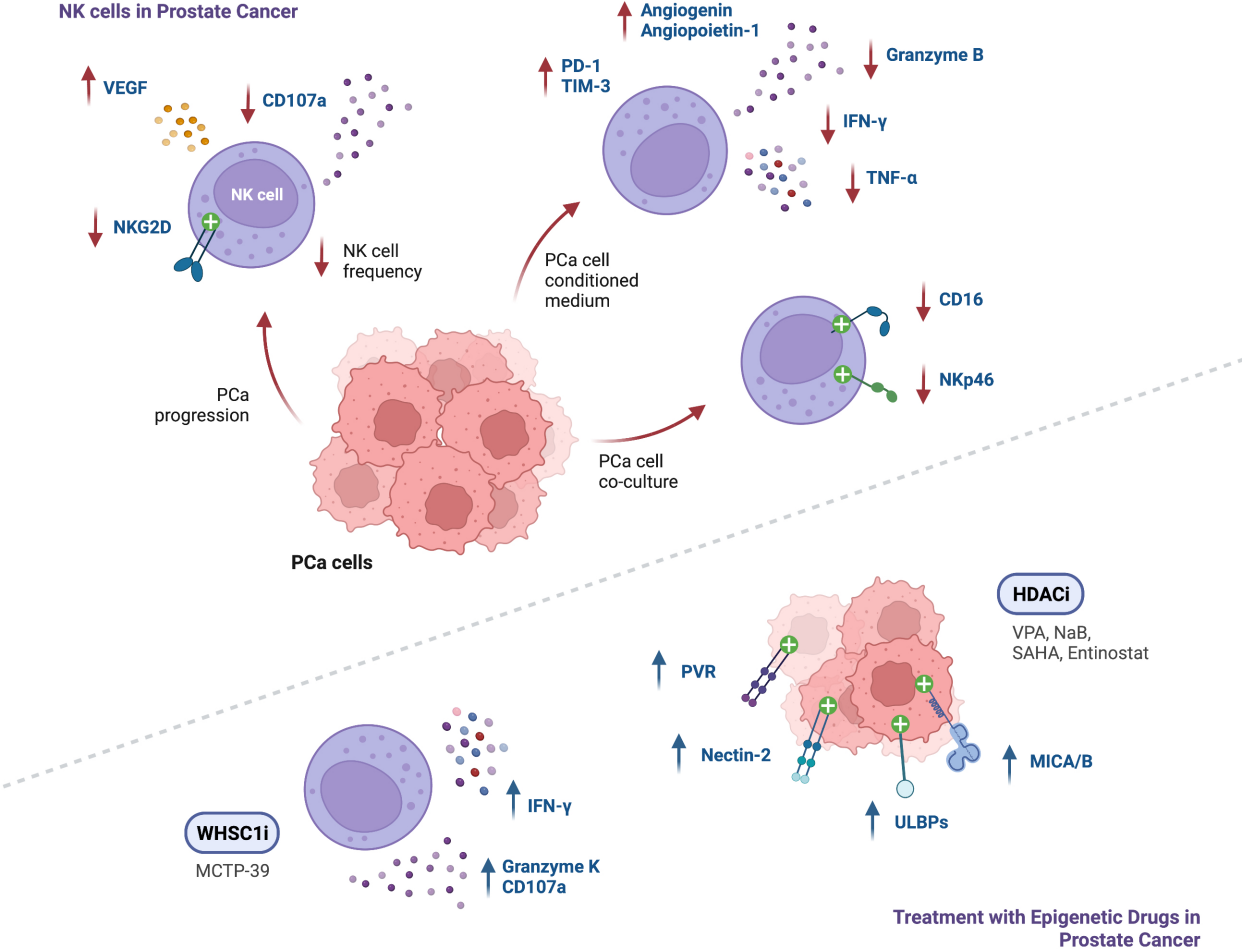
Summary of findings regarding NK cell function in the context of PCa, and modulation of NK cell anti-tumor responses following treatment with epigenetic modulating agents (↑ - upregulation; ↓ - downregulation).

The understanding of the immune landscape in prostate cancer and its impact on patient prognosis is increasing, however insights on the mechanisms involved in its regulation are still lacking. NK cells in PCa have shown increased expression of exhaustion markers PD-1 and TIM-3, impairing their degranulation ability and reducing production of TNF-α, IFN-γ and Granzyme-B. In addition, in PCa, NK cells seem to decrease in frequency, also displaying fewer activating receptors NKG2D, CD16 and NKp46 and impaired anti-tumor activity (**Figure 3**). These findings highlight the potential and need for additional studies on targeting NK cell-mediated immunity in PCa patients.

Epigenetic modulation of anti-tumor responses can be achieved through exposure to several pharmacological agents. Numerous studies conducted in several tumor models confirmed upregulation of NK cell activating ligands after cancer cell treatment with DNMT, HDAC, HMT, KDM and BET inhibitors, leading to enhanced NK cell cytotoxicity following co-culture with tumor cells. To date, in prostate cancer, it has been shown upregulation of NK-cell activating ligands such as MICA, MICB, ULBPs, PVR and Nectin-2 upon treatment with HDAC inhibitors such as VPA (alone or in combination with the DNMT inhibitor hydralazine), NaB, SAHA and entinostat (**Figure 3**). Hence, studies with other epigenetic modulating drugs in PCa are still lacking. Of note, it has been reported in other cancers, upregulation of the inhibitory ligand MHC-I after exposure to some epigenetic agents, including DNMTi, HDACi, EZH2i and G9a inhibitors. Moreover, downregulation of the activating NKp30 ligand, B7-H6, was observed upon treatment with HDACi and HATi. Thus, it should be considered the effect that an epigenetic drug might have in the complex balance between expression of activating and inhibitory ligands.

Several studies in tumor models other than PCa demonstrated that direct modulation of NK cells with different epigenetic agents can drive increased expression of activating receptors such as NKG2D, NKp30, and DNAM-1. Conversely, decreased expression of NKG2D, NKp46 and CD16, has also been shown after treatment with some HDACi, DNMTi, and LSD1i. Moreover, expression of inhibitory receptors, such as KIRs, was upregulated following NK cell treatment with entinostat and 5-aza-dC. The intricacy of these findings suggests that different agents can have different effects on NK cells, supporting the need of further research. In PCa, NK cell receptor modulation effect on tumor cell recognition with epigenetic agents is still unknown.

NK cell activity was found to be enhanced after treatment with agents such as entinostat, panobinostat, NextA, 5-aza, 5-aza-dC, MCTP-39, and EZH2 inhibitors, as demonstrated by increased expression of Perforin and Granzyme-B, and enhanced secretion of TNF-α and IFN-γ, which resulted in increased NK cell-killing of tumor cells. In fact, PCa mice xenografts treated with MCTP-39 showed increased NK cell effector function, as well as upregulation of IFN-γ and Granzyme-K (**Figure 3**).

Several NK cell-based immunotherapies have been studied and some are currently undergoing clinical trials for cancer treatment. These approaches include autologous and allogenic NK cell transfer following *ex vivo* expansion and activation with cytokines, NK cells derived from induced pluripotent stem cells (iPSCs), and genetically modified chimeric antigen receptor (CAR) NK cells targeting specific antigens on the surface of tumor cells (163). However, limited infiltration, immunosuppressive TME, and downregulation of tumor antigens and NK ligands, are factors restraining the clinical efficacy of those approaches (163, 164). Thus, we propose that combination with epigenetic modulating agents may represent a promising strategy to improve the effectiveness of NK cell-based immunotherapy.

Overall, despite some challenges, emerging data suggests exciting potential in modulating NK cell-mediated immunity. The therapeutic usage of epigenetic modulating agents for prostate cancer in order to render tumor cells more immunogenic and increase NK cell anti-tumor potential, is an exciting possibility to be pursued, opening new venues for further investigation.

## Author contributions

FDdR and MPC designed and wrote the manuscript. MPC and CJ revised the paper. All authors contributed to the article and approved the submitted version.
